# Supplementary material for: Comparative Proteomics of Marinobacter sp. TT1 Reveals Corexit Impacts on Hydrocarbon Metabolism, Chemotactic Motility, and Biofilm Formation
Source: Microorganisms. 2020 Dec 22;9(1):3. doi: 10.3390/microorganisms9010003 (PMC7822026; doi:10.3390/microorganisms9010003)
Supplement: Supplementary file 1 [file microorganisms-09-00003-s001.zip › Supplementary/Rugho╠êft_SupplementaryFigures.pdf]

Supplementary Material

Comparative proteomics of *Marinobacter* sp. TT1 reveals Corexit impacts on hydrocarbon metabolism, chemotactic motility and biofilm formation

Saskia Rughöft [1], Nico Jehmlich [2], Tony Gutierrez [3] and Sara Kleindienst [1,*]

[1] Microbial Ecology, Center for Applied Geosciences, University of Tübingen, Tübingen, Germany
[2] Department of Molecular Systems Biology, Helmholtz Centre for Environmental Research –UFZ GmbH, Leipzig, Germany
[3] Institute of Mechanical, Process & Energy Engineering, Heriot-Watt University, Edinburgh, UK

* Correspondence: sara.kleindienst@uni-tuebingen.de

Supplementary Figures

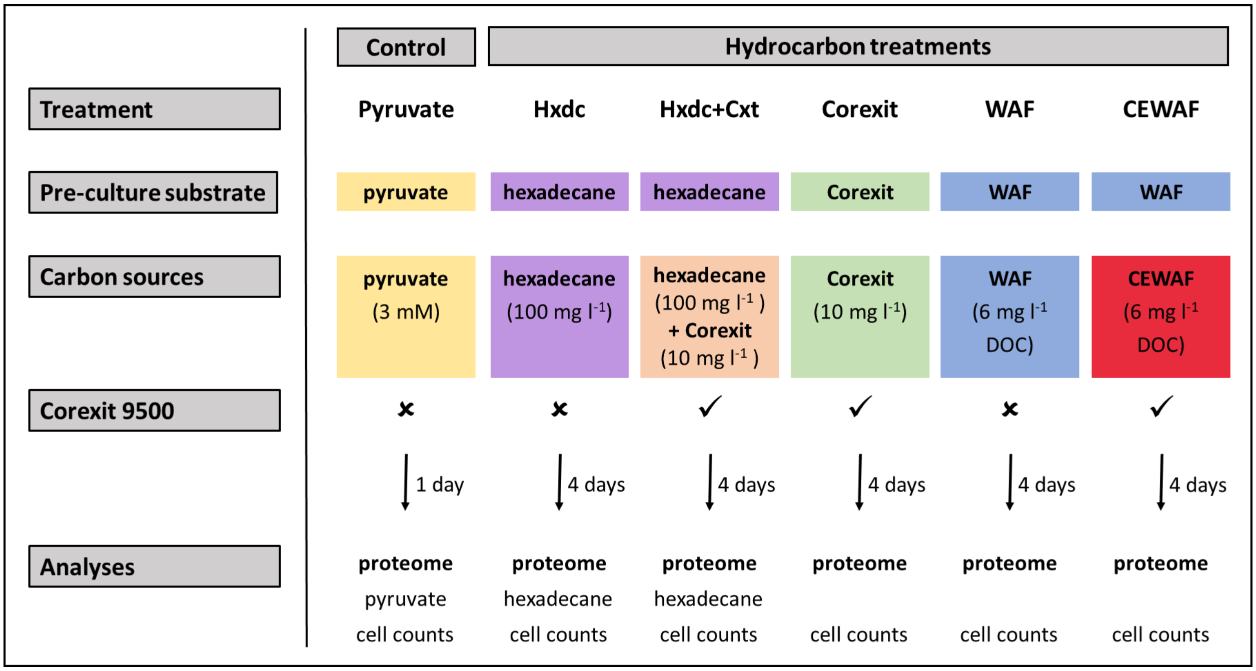

Figure S1: Overview of culture conditions assessed in this proteomics study. Additional non-proteomics treatments included an inoculated control culture with no added substrate and abiotic controls containing either 100 mg l^{-1} *n*-hexadecane or 100 mg l^{-1} *n*-hexadecane and 10 mg l^{-1} Corexit. Abbreviations: Hxdc = *n*-hexadecane, Cxt = Corexit EC9500A, WAF = water-accommodated fraction, CEWAF = chemically enhanced WAF, DOC = dissolved organic carbon.

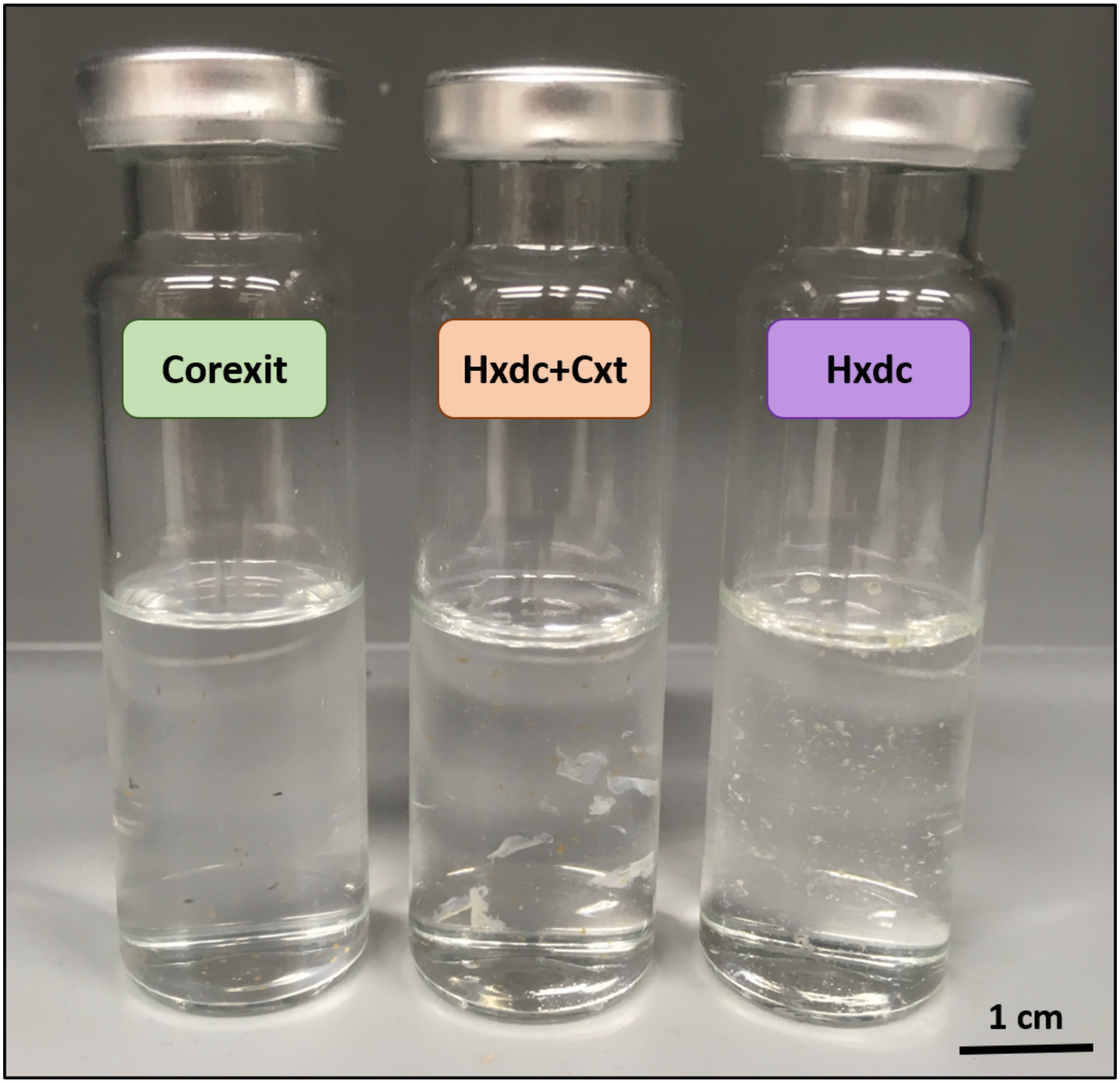

Figure S2: Aggregate morphology in *Marinobacter* sp. TT1 cultures after four days of incubation. Treatments contained the following carbon sources: 100 mg l^{-1} Corexit (Corexit), 100 mg l^{-1} *n*-hexadecane and 10 mg l^{-1} Corexit (Hxdc+Cxt), or 100 mg l^{-1} *n*-hexadecane (Hxdc).

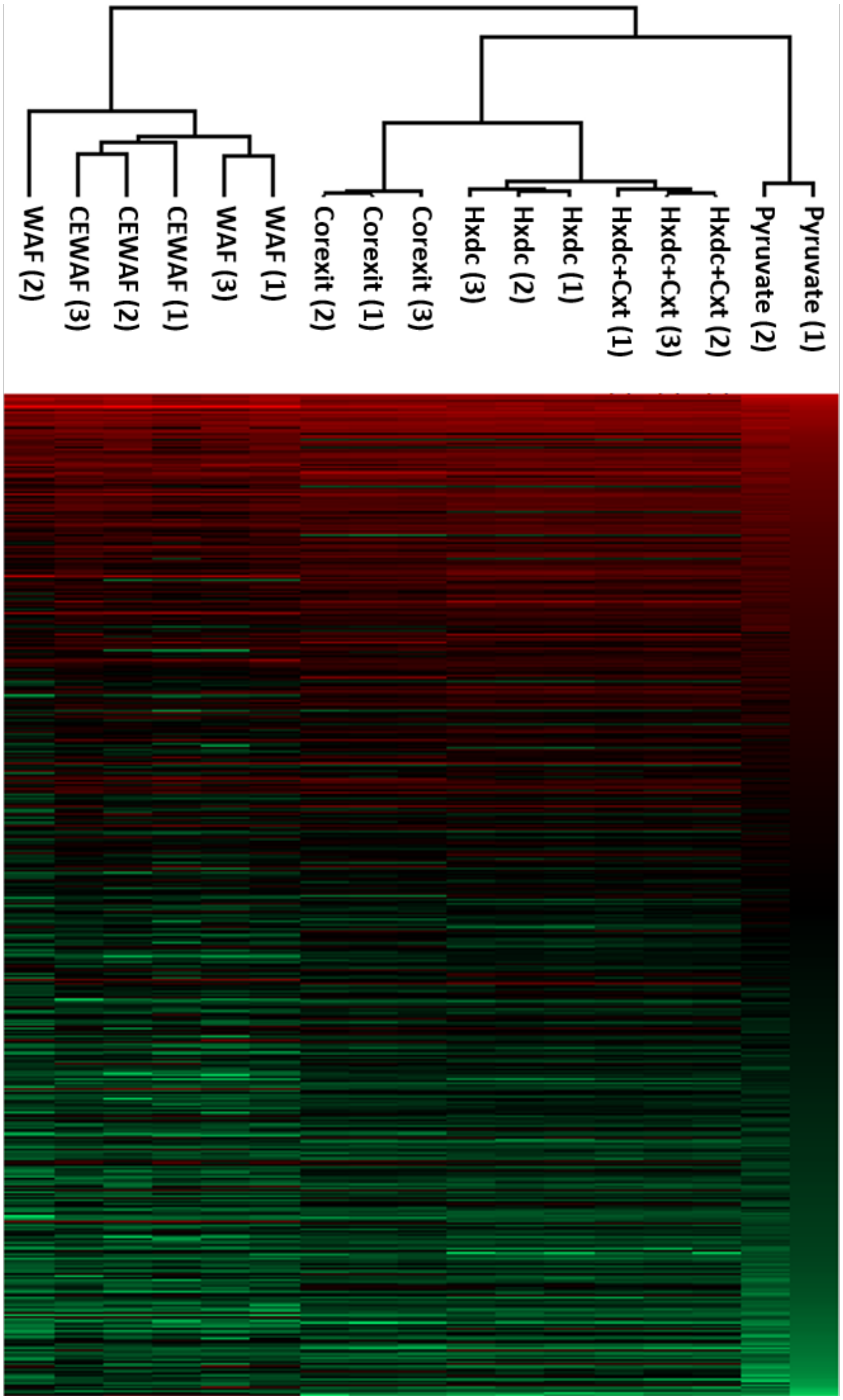

Figure S3: Hierarchical clustering analysis (including heatmap) of protein expression profiles of *Marinobacter* sp. TT1 cultures after 1 day (pyruvate treatments) or 4 days (all other treatments) of incubation. Treatments received the following carbon sources: 3 mM (264 mg l^{-1}) pyruvate (Pyruvate), 100 mg l^{-1} *n*-hexadecane (Hxdc), 100 mg l^{-1} *n*-hexadecane and 10 mg l^{-1} Corexit (Hxdc+Cxt), 100 mg l^{-1} Corexit (Corexit), 6 mg l^{-1} WAF-derived DOC (WAF), or 6 mg l^{-1} chemically enhanced WAF-derived DOC (CEWAF).

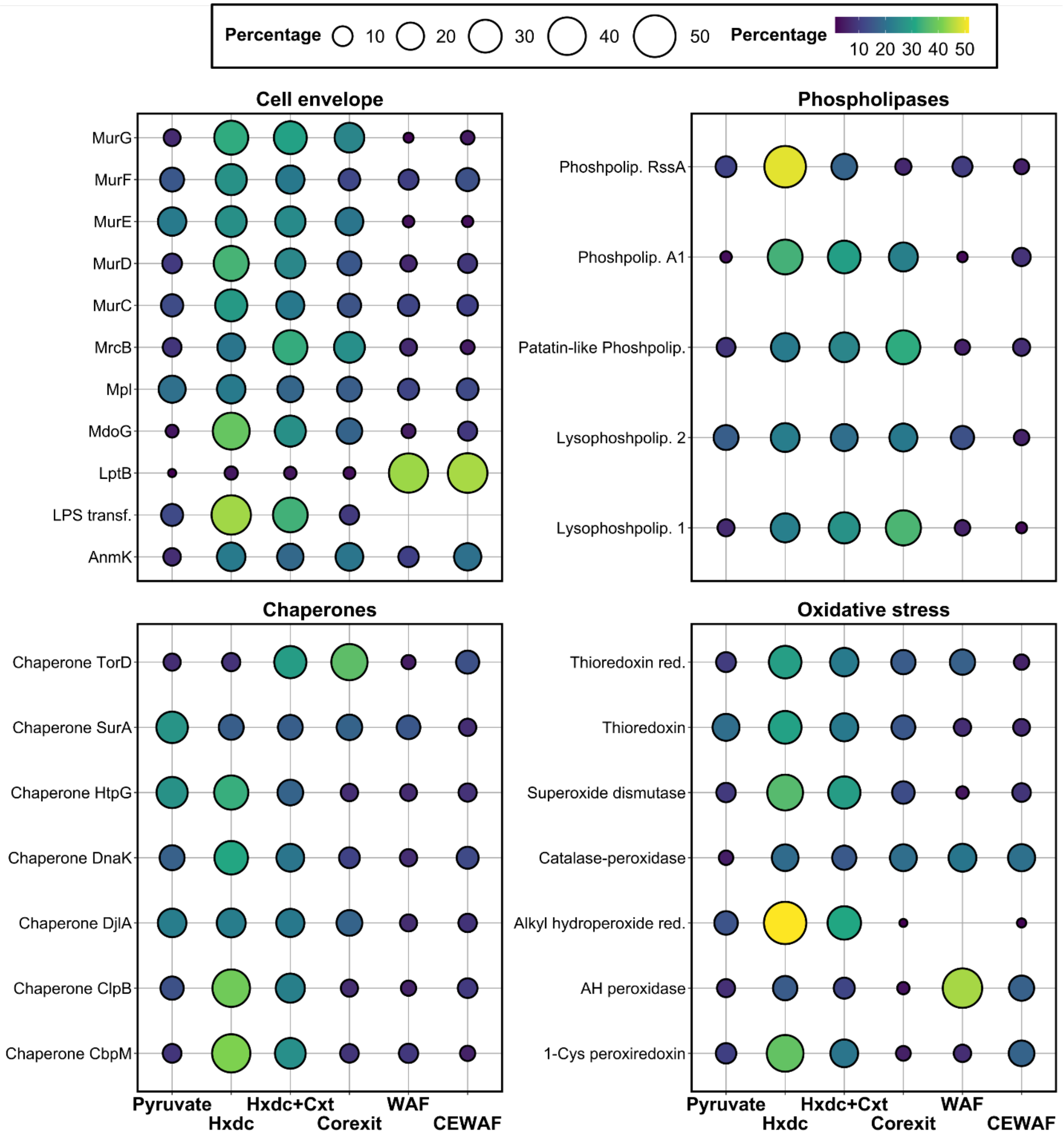

Figure S4: Normalized, relative mean abundances (symbolized by circle size and colour; sum per protein = 100%) of significantly (q-value < 0.05) differentially expressed proteins associated with peptidoglycan and LPS synthesis (Cell envelope), phospholipases, chaperones, oxidative stress response enzymes during growth of *Marinobacter* sp. TT1 cultures on different carbon sources. Treatments received either pyruvate, *n*-hexadecane (Hxdc), *n*-hexadecane and Corexit (Hxdc+Cxt), only Corexit (Corexit), crude oil WAF, or chemically enhanced WAF (CEWAF). Protein names/abbreviations given as indicated in Tab. S9.

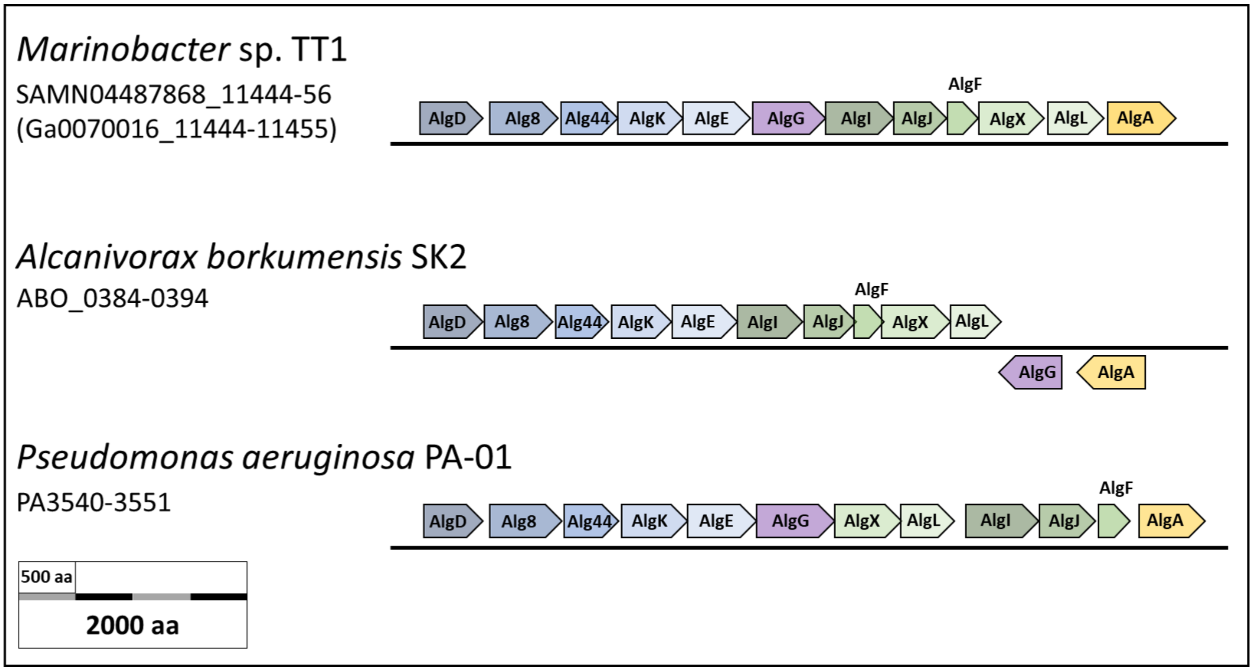

Figure S5: Genetic organization of alginate operons in *Pseudomonas aeruginosa* PAO1 (IMG genome ID: 637000218), *Alcanivorax borkumensis* SK2 (IMG genome ID: 637000004) and the proposed alginate operon in *Marinobacter* sp. TT1 (displayed gene names based on genome annotation and/or homologous *P. aeruginosa* genes; IMG genome ID: 2619618959).

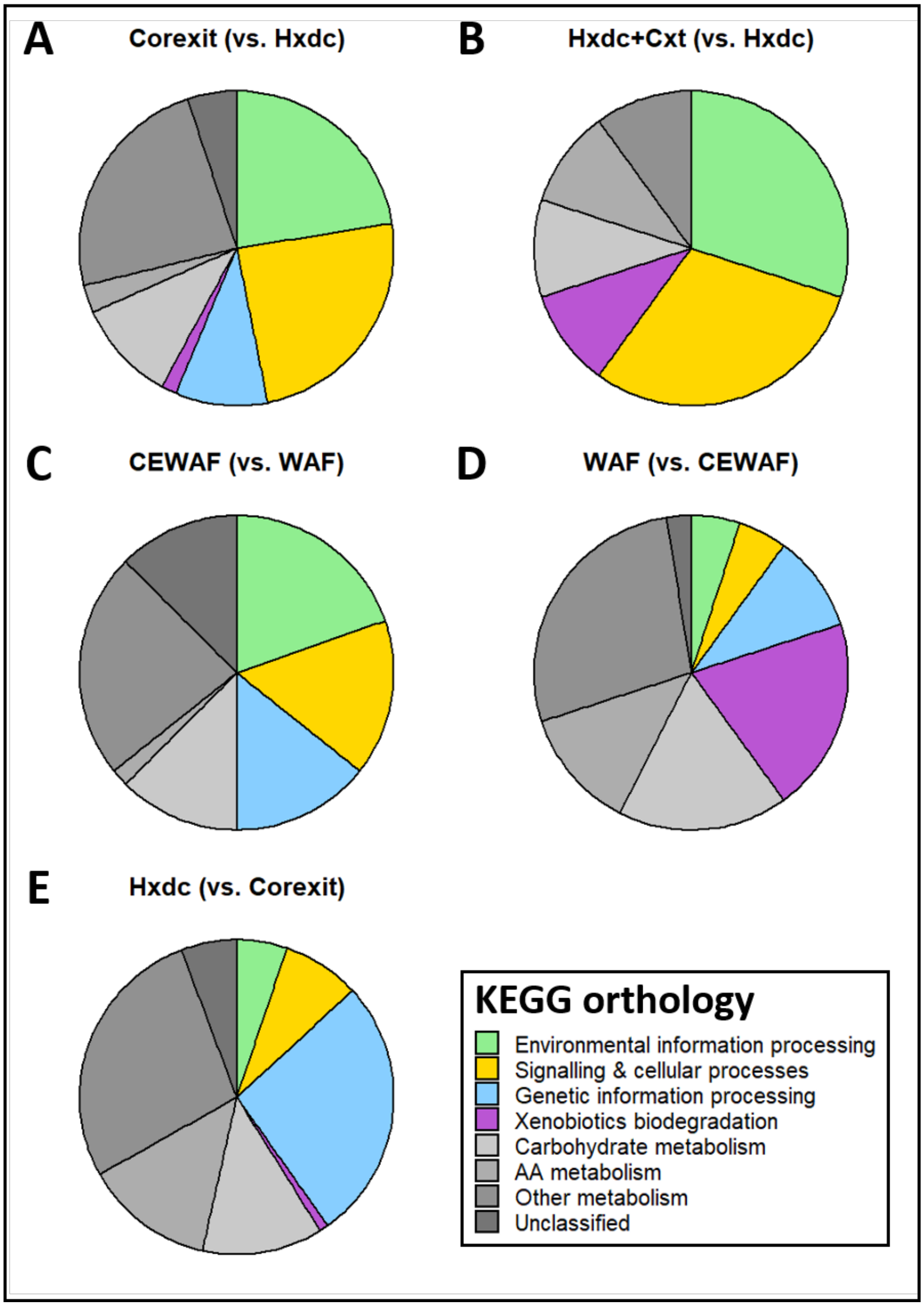

Figure S4: KEGG orthology annotations for significantly (p-value < 0.05; WAF vs. CEWAF: t-test p-value < 0.05) more abundant proteins in pairwise comparisons of *Marinobacter* sp. TT1 proteomes during growth on different carbon sources. Treatments received either pyruvate, *n*-hexadecane (Hxdc), *n*-hexadecane and Corexit (Hxdc+Cxt), only Corexit (Corexit), crude oil WAF, or chemically enhanced WAF (CEWAF). **A)** Significantly upregulated proteins in Corexit compared to Hxdc cultures. **B)** Significantly upregulated proteins in Hxdc+Cxt compared to Hxdc cultures. **C)** Significantly upregulated proteins in CEWAF compared to WAF cultures. **D)** Significantly upregulated proteins in WAF compared to CEWAF cultures. **E)** Significantly upregulated proteins in Hxdc compared to Corexit cultures.

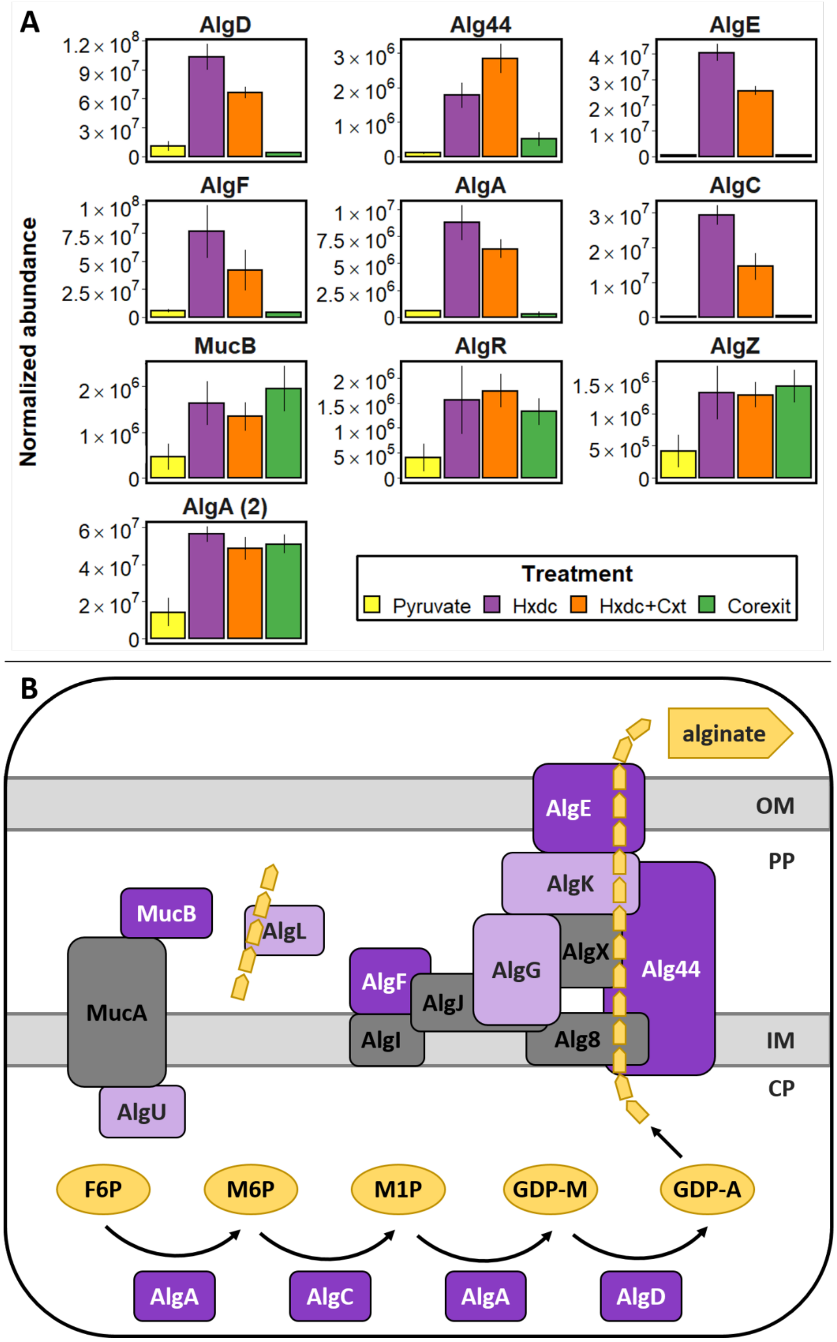

Figure S5: Alginate biosynthesis and export in *Marinobacter* sp. TT1. **A)** Normalized abundances (averages ± SD; n = 3) of significantly (q-value < 0.05) differentially abundant alginate protein homologues during growth on different carbon sources. Treatments received 3 mM (264 mg l^{-1}) pyruvate (Pyruvate), 100 mg l^{-1} *n*-hexadecane (Hxdc), 100 mg l^{-1} *n*-hexadecane and 10 mg l^{-1} Corexit (Hxdc+Cxt), or 100 mg l^{-1} Corexit (Corexit). **B)** Schematic overview of the proposed alginate biosynthesis complex in *Marinobacter* sp. TT1 based on the alginate complex in *Pseudomonas aeruginosa* (Hay *et al.*, 2013). Proteins are coloured in dark purple, light purple or grey to illustrate significantly higher abundances in *n*-hexadecane compared to pyruvate treatments, their detection or lack thereof in this study, respectively. Protein names given as indicated in Tab. S9. OM/IM = outer/inner membrane; PP/CP = periplasm/cytoplasm; F6P = Fructose-6-phoshate; M6P/M1P = Mannose-6-phosphate/-1-phosphate; GDP-M/A = Guanosine diphosphate-mannose/mannuronic acid.

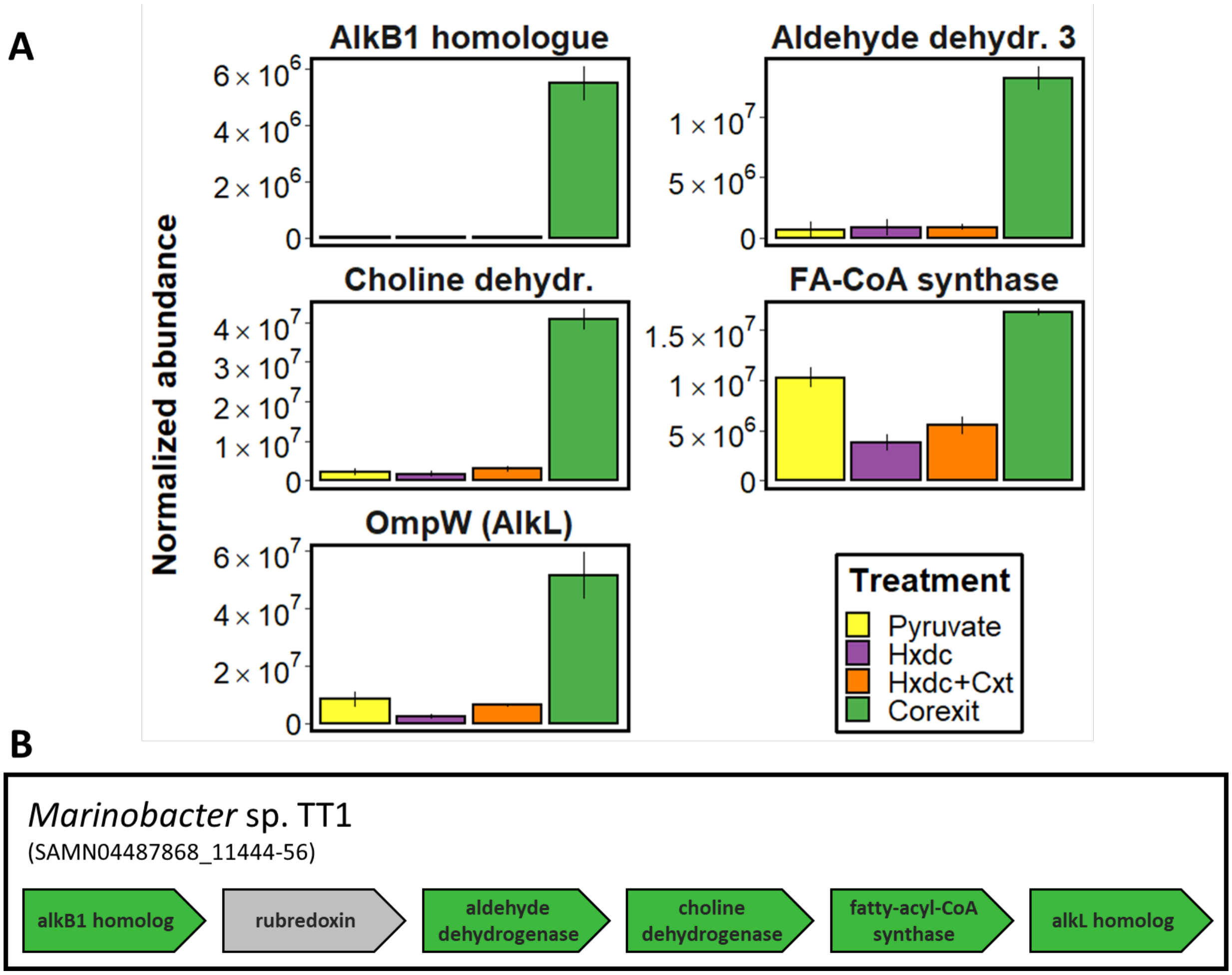

Figure S6: Proposed *alk* operon in *Marinobacter* sp. TT1. **A)** Normalized abundances (averages ± SD; n = 3) of significantly ($q < 0.05$) differentially expressed proteins of the *alkB1* homologue operon during growth on different carbon sources. Treatments received either pyruvate, *n*-hexadecane (Hxdc), *n*-hexadecane and Corexit (Hxdc+Cxt), or only Corexit (Corexit). **B)** Schematic representation of the genes downstream of *alkB1* (proposed *alk* operon; SAMN04487868_109138-42) in the genome of *Marinobacter* sp. TT1. Proteins encoded by green genes were significantly more abundant in Corexit treatments compared to Pyruvate, Hxdc or Hxdc+Cxt treatments. Proteins encoded by grey genes were not detected in this study. Protein names given as indicated in Tab. S9.

References

Hay, I.D., Ur Rehman, Z., Moradali, M.F., Wang, Y., and Rehm, B.H. (2013) Microbial alginate production, modification and its applications. *Microb Biotechnol* **6**(6): 637-650.
